# Critical Role of Macrophages and Their Activation via MyD88-NFκB Signaling in Lung Innate Immunity to *Mycoplasma pneumoniae*

**DOI:** 10.1371/journal.pone.0014417

**Published:** 2010-12-23

**Authors:** Jen-Feng Lai, Carlene L. Zindl, Lynn B. Duffy, T. Prescott Atkinson, Yong Woo Jung, Nico van Rooijen, Ken B. Waites, Duncan C. Krause, David D. Chaplin

**Affiliations:** 1 Department of Microbiology, University of Alabama at Birmingham, Birmingham, Alabama, United States of America; 2 Department of Pathology, University of Alabama at Birmingham, Birmingham, Alabama, United States of America; 3 Department of Pediatrics, University of Alabama at Birmingham, Birmingham, Alabama, United States of America; 4 Department of Molecular Cell Biology, Vrije University Medical Center, Amsterdam, The Netherlands; 5 Department of Microbiology, University of Georgia, Athens, Georgia, United States of America; Duke University Medical Center, United States of America

## Abstract

*Mycoplasma pneumoniae* (*Mp*), a common cause of pneumonia, is associated with asthma; however, the mechanisms underlying this association remain unclear. We investigated the cellular immune response to *Mp* in mice. Intranasal inoculation with *Mp* elicited infiltration of the lungs with neutrophils, monocytes and macrophages. Systemic depletion of macrophages, but not neutrophils, resulted in impaired clearance of *Mp* from the lungs. Accumulation and activation of macrophages were decreased in the lungs of MyD88^−/−^ mice and clearance of *Mp* was impaired, indicating that MyD88 is a key signaling protein in the anti-*Mp* response. MyD88-dependent signaling was also required for the *Mp*-induced activation of NFκB, which was essential for macrophages to eliminate the microbe *in vitro*. Thus, MyD88-NFκB signaling in macrophages is essential for clearance of *Mp* from the lungs.

## Introduction

Asthma is a chronic inflammatory disease of the airways, driven by Th2 lymphocytes and the type II cytokines IL-4, IL-5 and IL-13. These signals lead to the recruitment of neutrophils, monocytes, macrophages, lymphocytes, eosinophils and mast cells into the lung tissue and airway lumen. Infiltration of these cells into the lungs is associated with high-level production of airway mucus and the development of airway hyper-reactivity [1].

Typical of most complex diseases, asthma susceptibility appears to be multifactorial, including contributions by several genes and multiple environmental factors. Genetic susceptibility involves genes encoding functionally and structurally defined families of molecules that are thought to determine risk of both atopy and asthma. At least three groups of related genes have shown linkage to asthma susceptibility: genes that govern innate immune responses to environmental threats (CD14, TLR2, TLR4, TLR6, NOD1 and NOD2); genes involved in differentiation and activation of Th2 cells (IL-4, IL-13, IL-4R and GATA3); and genes that activate a broad range of inflammatory functions including recruitment of leukocytes to epithelial and endothelial surfaces (TNF, LTα, CCL5, CCL24 and CCL26) [2].

In addition to genetic susceptibility, environmental factors, including microbes, allergens and drugs, appear to contribute either to the induction or to increased severity of existing asthma [3]. One of the asthma-associated pathogens is *Mycoplasma pneumoniae* (*Mp*) [4], [5], [6]. In 1998, Kraft and colleagues showed, using PCR analysis of bronchoalveolar lavage (BAL) fluid, that more than 40% of patients with chronic stable asthma, compared to less than 10% of healthy control subjects, were positive for *Mp* in their airways [7]. Antibiotic treatment reduced the severity of asthma symptoms and improved pulmonary function in the subset of asthmatic patients whose BAL fluids or endobronchial biopsies were positive for *Mp*, whereas asthmatic patients who were *Mp* negative showed no improvement with antibiotic treatment [8]. Although not all studies have detected a higher rate of recovery of *Mp* from the airways of asthmatic subjects compared to controls [9], these combined observations suggest that the presence of microbes such as *Mp* in the airways may contribute to exacerbation of asthma symptoms in many populations.

Mycoplasmas are the smallest known self-replicating microorganisms. They are primarily mucosal pathogens, residing extracellularly in close association with epithelial surfaces. *Mp* is a human pathogen that typically infects ciliated epithelial cells in the respiratory tract, producing upper and lower airway infection [10]. Because *Mp* lacks all of the genes involved in amino acid synthesis, it is dependent on an exogenous supply of amino acids from its environment. Thus, *Mp* depends on a close association with host cells for its survival. The importance of this close association is underscored by the essential role of the *Mp* attachment organelle for microbial virulence. This specialized structure, located at the leading end of the bacterium, mediates close physical interactions between *Mp* and host epithelial cells. The attachment organelle of *Mp* is also essential for cell division and gliding motility [11]. The P1 and P30 adhesins, two critical components of this attachment organelle, play fundamental roles in the interactions of *Mp* with host airway epithelial cells and are required for virulence [10], [12], [13], [14].

Pattern recognition receptors (PRR) are essential for innate immunity to invading pathogens. One of the best-characterized groups of PRR is the family of Toll-like receptors (TLR). Signal transduction in the TLR pathways is mediated by four activating adaptors, including myeloid differentiation factor 88 (MyD88), TIR domain-containing adaptor protein (TIRAP, also called MyD88 adaptor-like (MAL)), TIR domain-containing adaptor inducing interferon-β (TRIF, also known as toll-IL-1 receptor adaptor molecule 1 (TICAM-1)), and TRIF-related adaptor molecule (TRAM, also named toll-like receptor adaptor molecule 2 (TICAM-2)) [15], [16]. MyD88 serves as the major adaptor molecule for all TLRs, except TLR3. TLR signals transduced through MyD88 ultimately activate NFκB and several of the interferon regulatory factors, all of which are transcription factors that regulate the expression of downstream genes required for TLR-induced cell activation. These downstream genes encode MHC molecules, T cell costimulatory molecules, cytokines, chemokines and their receptors, and many other activation associated genes [15], [17], which are critical for immune cells to respond to and eliminate invading pathogens.

Recent studies using experimental infection of mice have shown that IL-12 modulates the host response to *Mp* [18] and have established that signaling by TLR2 is required for the normal increased production of mucus by *Mp*-challenged airway epithelial cells [18], [19]. Nevertheless, the cellular mechanisms used by the host to control *Mp in vivo* remain largely undefined.

For this study, we developed a sensitive, specific and rapid method to detect *Mp* quantitatively in mouse lungs by using real-time PCR and verified that this assay detected similar numbers of *Mp* in the lungs of experimentally infected mice compared to quantitation by colony count of *Mp* recovered from minced lung tissue. Using this method, we focused on the cellular and molecular mechanisms used by the host to clear *Mp* from the lungs in an experimental mouse model. We show that macrophages, but not neutrophils, play a critical role in clearance of *Mp* from the lungs of mice. Additionally, we demonstrate an essential role for MyD88-NFκB signaling in the macrophage response to *Mp in vivo* and *in vitro*. This signaling pathway is required both for activation of macrophages in the lungs and for elimination of *Mp* both from the airways and from cultures of bone marrow-derived macrophages.

## Results

### Quantitative detection of *Mp* in the lungs of mice

*Mp* can be cultured on SP4 agar, but replicates slowly. Because of its lack of a cell wall and limited capacity to synthesize many important biomolecules, *Mp* is very sensitive to changes in its microenvironment. In addition, recent studies suggest that *Mp* may be a facultative intracellular pathogen, from which compartment it may be difficult to culture or affect with certain classes of antibiotics [20]. To quantify *Mp* rapidly from infected tissues, we developed a real-time PCR-based method for measuring the numbers of *Mp* in extracts of mouse lung.

For the real-time PCR assay we targeted the *Mp* 16S rRNA, because portions of this RNA are unique for each of the known mycoplasma strains and because the RNA is present in high copy number in the microbe. The assay is focused on the variable 5′ end region of the *Mp* 16S rRNA. This portion of the *Mp* 16S rRNA gene has been used as a target for a DNA-based semi-quantitative PCR assay in the past [21], but not using the RNA-based real-time PCR technology. We used the murine housekeeping gene, GAPDH, as an internal control for normalization of the amount of lung tissue represented in each RNA preparation.

**Figure 1:**
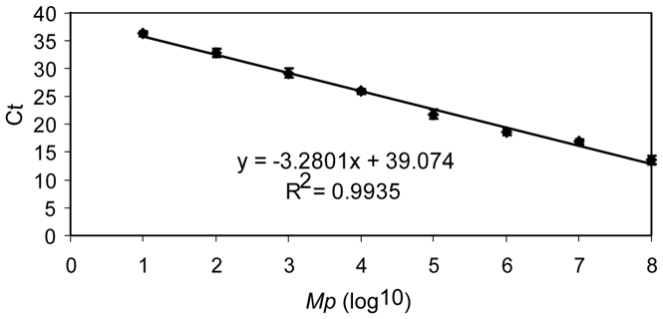
Quantitative detection of *Mp* in mouse lung by real-time PCR. A portion of the 16S rRNA (from nucleotides 201 to 265) that is specific for *Mp* was chosen as the molecular target for detection. Total RNA was purified from the lungs of one mouse, and RNA from the indicated number of *Mp* was added. 100 ng of total RNA was used for each real-time PCR reaction. Ct, cycle threshold. The sensitivity and linearity of the assay were confirmed in one additional experiment. Data shown are means ± SEM.

In order to generate a standard curve for numbers of *Mp*, total RNA was purified from the lungs of one non-infected mouse, and RNA from the indicated numbers (1 to 10^8^) of *Mp* was added. 100 ng of total RNA was used for each real-time PCR reaction. Over a range of <10^2^ to 10^8^ mycoplasmas per lung, this assay showed a linear relationship between the numbers of *Mp* (x-axis, log_10_) and the threshold number of cycles (y-axis, Ct) (Figure 1). Thus, this assay provides a detection sensitivity of <100 *Mp* in the lungs of an individual mouse. In order to assess the degree to which real-time PCR and bacterial culture with colony count yield comparable data, we analyzed *Mp* numbers in the lungs of mice using both techniques in the same experiment. Our data showed that real-time PCR and conventional culture and colony count provided very similar numbers of *Mp* in the lungs of infected mice at d1, d3, and d5 after inoculation with *Mp* (Figure S1). Because the real-time PCR assay is more rapid and equally sensitive compared to bacterial culture, we have used it for the remainder of our studies.

### Time course for the clearance of *Mp* from the lungs of C57BL6 and BALB/c mice

**Figure 2:**
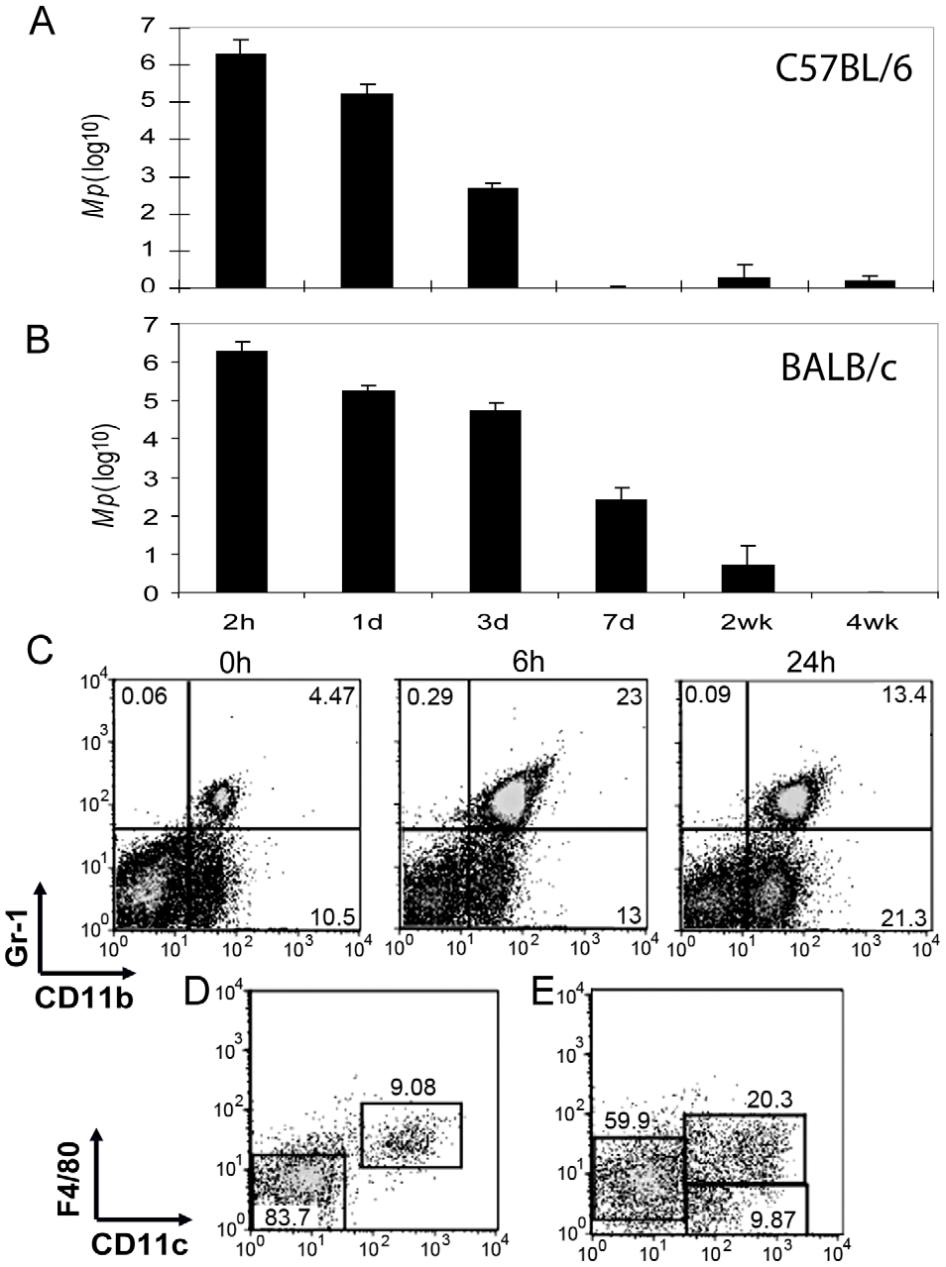
Host responses to *Mp*. Mice were inoculated intranasally with 4×10^6^ *Mp*. At the indicated times, total lung RNA was harvested and the numbers of *Mp* were measured by real-time PCR. Time course of *Mp* clearance from lungs of C57BL/6 mice (A) and BALB/c mice (B). For examining the cellular host response to respiratory *Mp*, whole lungs were digested into single cell suspensions and stained with Abs for FACS analysis. (C) At 6 h and 24 h after infection with *Mp*, CD11b^+^Gr-1^hi^ and CD11b^+^Gr-1^lo/−^ cell populations were recruited in the lungs. (D) Within the CD11b^+^Gr-1^hi^ population of cells analyzed 24 h post-*Mp* infection, the major cell types were neutrophils (CD11c^−^F4/80^−^) and a small subset of pulmonary macrophages (CD11c^+^F4/80^+^). (E) The CD11b^+^Gr-1^lo/−^ cell population includes pulmonary macrophages (CD11c^+^F4/80^+^), macrophage/monocytes (CD11c^−^F4/80^lo/−^) and DC (CD11c^+^F4/80^−^ ). Each experiment was replicated at least two times. Data shown are means ± SEM (n = 6).

Mice were inoculated i.n. with 4×10^6^ *Mp* on d0, and were sacrificed 2 h, 1 d, 3 d, 7 d, 2 wk, and 4 wk later. Based on an analysis of the numbers of *Mp* using the real-time PCR assay, *Mp* were cleared from the lungs of C57BL/6 mice within 7 days post-inoculation (Figure 2A); in BALB/c mice, the clearance of *Mp* from the lungs was reproducibly slower, reaching numbers below the level of detection of the assay by 4 weeks indicating that there are genetic background effects on *Mp* clearance (Figure 2B). To eliminate this type of variability, for the remainder of our study we used C57BL/6 as the wild type (WT) mouse strain. Importantly, in both strains of mice, 90% or more of the *Mp* were eliminated from the lungs over the first day (Figures 2A and 2B). This rapid, early clearance of *Mp* suggested that innate host responses play a major role in the elimination of *Mp* from the lungs of mice. We tested the impact of varying the inoculating dose of bacteria over a range from 4×10^5^ to 4×10^7^ cfu/animal. At days 2 and 3 after inoculation, the numbers of *Mp* recovered from the lungs were in proportion to the infectious dose, indicating that the ability of these innate responses to handle the bacterial load were not saturated at this level of challenge (data not shown).

Consistent with this, there were no significant differences in the rate of clearance of *Mp* from the lungs of WT mice and Rag1^−/−^ mice, which lack T and B lymphocyte-based adaptive immune responses [22] (Figure S2). These data support the central importance of the innate immune response in the elimination of *Mp* from the lungs of mice.

### Rapid recruitment of neutrophils and macrophages/monocytes to the mouse lung following inoculation with *Mp*

To investigate the role of innate cells in the clearance of *Mp*, we first determined the nature of the host's cellular inflammatory response following airway inoculation with the microbe. Leukocytes that can be recovered from the lungs of microbe-challenged animals consist both of resident populations of innate cells and additional cells recruited from the periphery in response to the potential pathogen. The resident cell populations include macrophages, monocytes, dendritic cells (DC), NK cells, mast cells, neutrophils and other granulocytes, all cell types that can respond to invading pathogens.

Following i.n. inoculation with *Mp*, there was a rapid and substantial increase in the numbers of CD11b^+^ cells in the lungs (Figure 2C). Based on their staining with anti-Gr-1, these CD11b^+^ cells comprised two subpopulations, CD11b^+^Gr-1^hi^ and CD11b^+^Gr-1^lo/−^. By 6 hr, the CD11b^+^Gr-1^hi^ cells were dramatically increased from <5% of total lung leukocytes to >20% of total lung leukocytes. This population remained elevated at >10% of total lung cells at 24 h after inoculation with *Mp* (Figure 2C). The CD11b^+^Gr-1^lo/−^ cells were increased from 10.5% of total lung cells in control animals to 13% of total cells 6 hr after inoculation with *Mp*, and to >20% by 24 h after microbial challenge (Figure 2C). The numbers of both of these two cell populations had returned to the baseline by 2–4 days after inoculation with *Mp* (data not shown).

Each of these CD11b^+^ cell populations could be further fractionated based on their surface expression of F4/80 (typically expressed on macrophages) and CD11c (typically expressed on DC and pulmonary macrophages). The largest cellular subpopulation, representing approximately 80–90% of the cells in the CD11b^+^Gr-1^hi^ gate, did not stain with antibodies to CD11c or F4/80 (Figure 2D), and expressed Ly6G, but not Ly6C (data not shown). Based on this pattern of cell surface marker expression, this subset has phenotypic characteristics typical of neutrophils [23]. A minority subpopulation in the CD11b^+^Gr-1^hi^ gate expressed CD11c and F4/80 (Figure 2D), typical of pulmonary macrophages [24].

Analysis of cells within the CD11b^+^Gr-1^lo/−^ gate for expression of CD11c and F4/80 discriminated three subpopulations (Figure 2E). Cells that were CD11b^+^Gr-1^lo/−^CD11c^−^F4/80^lo/−^ were monocytes/macrophages, whereas cells that were CD11b^+^Gr-1^lo/−^CD11c^+^F4/80^+^ were activated pulmonary macrophages. The minority subpopulation that was CD11b^+^Gr-1^lo/−^ CD11c^+^F4/80^−^ consisted primarily of myeloid-type DC [25], [26], [27].

Altogether, these data demonstrated that both neutrophils (CD11b^+^Gr-1^hi^CD11c^−^F4/80^−^) and monocytes/macrophages (CD11b^+^Gr-1^lo/−^CD11c^+/−^F4/80^+/lo^ or CD11b^+^Gr-1^hi^CD11c^+^F4/80^+^) were recruited into the lungs within the first 24 h after i.n. instillation of *Mp*, during the period when 90% or more of the bacteria were cleared from the lungs (Figures 1, 2C, 2D, and 2E).

### Clearance of *Mp* from the airways of mice is insensitive to depletion of neutrophils

**Figure 3:**
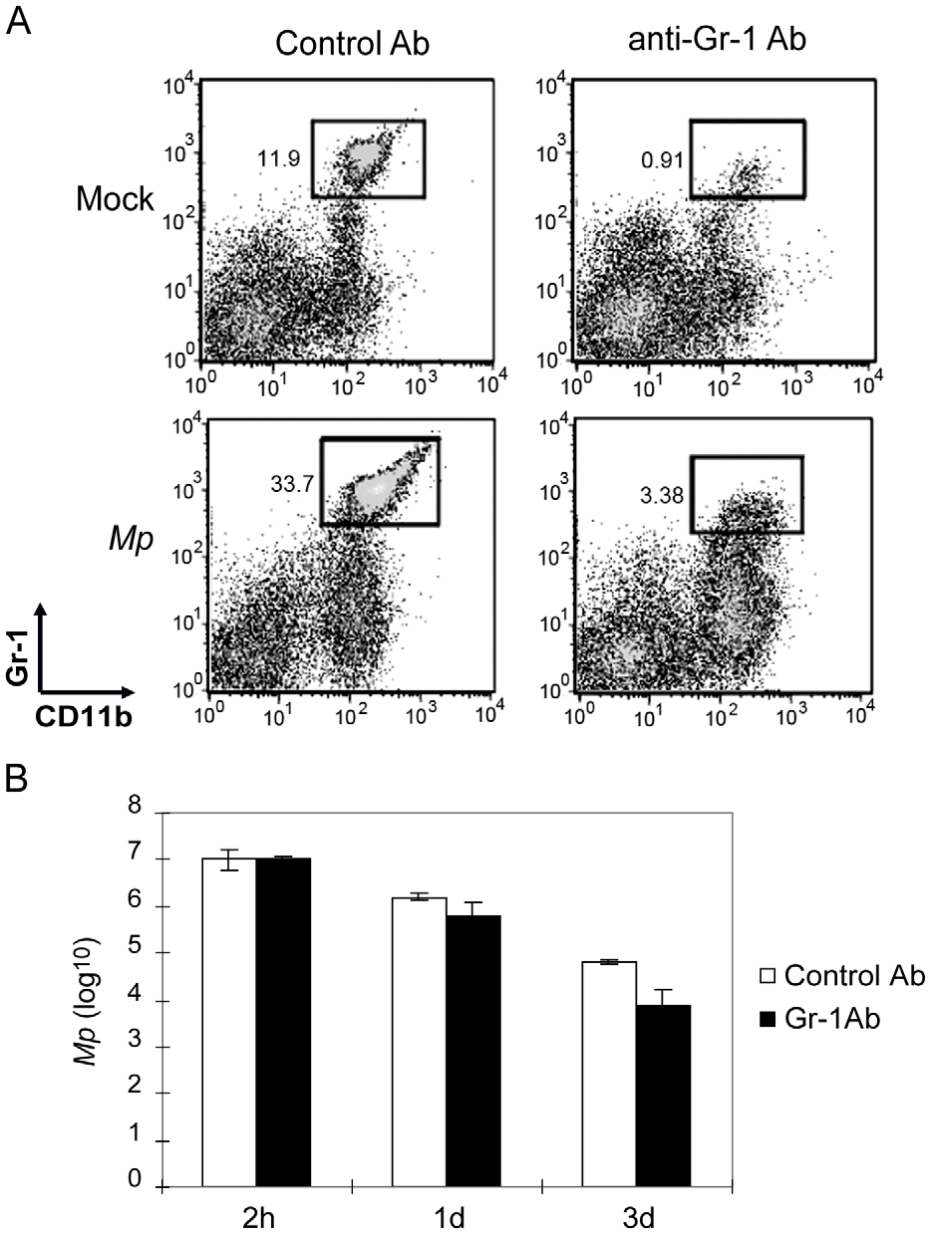
Neutrophils are not required for clearance of *Mp* from mouse lung. (A) Greater than 90% of the CD11b^+^Gr-1^hi^ cells (mainly neutrophils) in mouse lung were depleted by i.v. injection with anti-Gr-1 Ab (RB6-8C5) with or without *Mp* infection. Data shown were collected 3 d after injection of the anti-Gr-1 Ab, and 1 d after inoculation i.n. with *Mp*. (B) Neutrophil depleted mice (filled bars) showed no significant difference in clearance of *Mp* from the lungs at all indicated times post-*Mp* inoculation, compared to control mice (open bars). At least three similar experiments were performed with comparable results. Data shown are means ± SEM (n = 4 or 5). P>0.05, comparing control and Gr-1 Ab injected mice at each of the time points.

Because neutrophils were recruited rapidly and in high numbers into the lungs of mice following i.n. inoculation with *Mp* (Figures 2C and 2D), we tested whether this cell population contributed to the clearance of the organism from the lungs. We induced systemic depletion of neutrophils by treatment of adult WT mice with i.v. injection of 100 µg of an anti-Gr-1 mAb (clone RB6-8C5) prior to i.n. inoculation with *Mp*. Two days after injection with RB6-8C5, the depletion of CD11b^+^Gr-1^hi^ cells in both the blood and the lungs was greater than 99% [28]. There was no significant depletion of Gr-1^lo^ populations in either blood or lungs [28]. One day after inoculation with *Mp*, depletion of neutrophils remained at a high level in the anti-Gr-1 Ab-treated mice, with only 10% as many lung neutrophils as observed in microbe challenged mice that had received the control mAb (Figure 3A).

Neutrophil-depleted mice cleared *Mp* from their lungs at a rate equal to or modestly faster than mice treated with the control Ab (Figure 3B). In order to confirm that neutrophils are not essential for the clearance of *Mp* from the lungs, we tested CD11b^−/−^ mice, which have morphologically abnormal neutrophils, and impaired neutrophil recruitment, activation, and apoptosis [29], [30]. As with mice treated with the RB6-8C5 mAb, we found no difference in the clearance of *Mp* from lungs of CD11b^−/−^ mice compared to WT control animals (Figure S3). Together, these data indicate that neutrophils do not play an essential role for elimination of *Mp* from the lungs of naïve mice.

### Systemic depletion of macrophages impairs the elimination of *Mp* from the lungs

**Figure 4:**
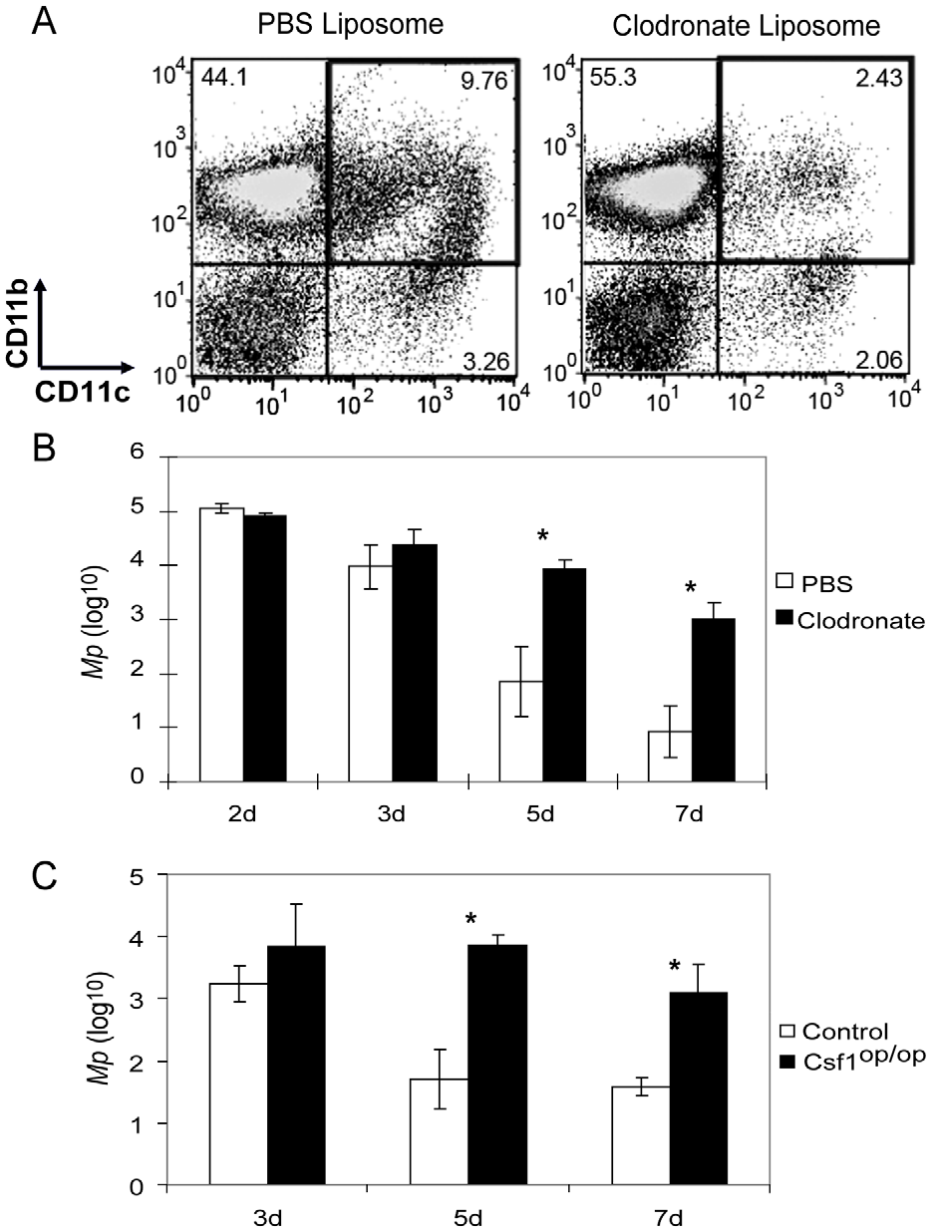
Macrophages play an essential role in the elimination of *Mp* from the lungs of mice. (A) Greater than 70% of the pool of CD11c^+^CD11b^+^ cells (mainly macrophages) in the lungs of *Mp-*challenged mice was depleted by injection (i.p. + i.n.) with clodronate-containing liposomes (3 d after injection with liposomes, 1 d after inoculation with *Mp*). (B) and (C) Macrophage-depleted mice (filled bars) (B) and CSF1^op/op^ mice (filled bars) (C) showed impaired clearance of *Mp* from the lungs at the indicated times post-*Mp* inoculation, compared to control mice (open bars). For clodronate depletion of macrophages, at least 5 experiments were performed with similar results. For CSF1^op/op^ mice, the experiment was repeated once with similar results. Data shown are means ± SEM (n = 3 or 5). * p<0.05.

To test the role of macrophages in the host responses to *Mp*, we depleted macrophages systemically by injection of clodronate-containing liposomes both i.p. and i.n. Injection of clodronate liposomes by both the i.n. and the i.p. routes was required to obtain effective depletion of both resident pulmonary (CD11c^hi^CD11b^lo^) and recruited and interstitial (CD11c^lo^CD11b^hi^) monocyte/macrophages following microbial challenge (Figure S4). One day after liposome injection, mice were inoculated i.n. with *Mp*. In animals that had been treated with the control liposomes, the percentage of CD11b^+^CD11c^+^ cells (mainly activated resident macrophages and recruited monocyte/macrophages, and a small subset of DC) was 9.76%. This cell population was substantially reduced (to <2.5%) in the lungs of mice that had been treated with clodronate liposomes prior to i.n. challenge with *Mp* (Figure 4A). This greater than 70% depletion in lung macrophages was associated with impaired clearance of *Mp* from the lungs. Numbers of *Mp* were not significantly different at days 2 and 3 after i.n. microbial challenge; however, at d 5 and d 7 after inoculation, there were several hundred-fold more *Mp* in the lungs of the clodronate liposome-treated mice compared to mice treated with the control liposomes (Figure 4B). These data show that, while macrophages are not essential for the initial elimination of *Mp* from lungs of mice observed in the first 3 days after microbial challenge of the airway, macrophages are essential for the final elimination of the microbe after d 3. Interestingly, varying the inoculating dose of *Mp* over a 100-fold range did not alter the ability of WT mice to clear the bacteria by d 5 (data not shown), suggesting that the ability of the respiratory tract macrophages to clear *Mp* remaining at these late time points is relatively independent of the initial bacterial load.

To confirm the importance of macrophages in the clearance of *Mp*, we also tested clearance from the lungs of Csf1^op/op^ mice, which manifest reduced numbers of macrophages and monocytes [31] because of a mutation in CSF1 (also designated M-CSF). The numbers of *Mp* recovered from the lungs of Csf1^op/op^ mice at 3 d after i.n. inoculation were not statistically different from the numbers recovered from control mice (either Csf1^op/+^ or Csf1^+/+^ mice) (Figure 4C); however, at d 5 and d 7 post-inoculation, there were 10- to 100-fold greater numbers of *Mp* in the lungs of Csf1^op/op^ mice compared to control animals (Figure 4C). Thus, when the numbers of host macrophages are reduced either due to a congenital deficiency in the production of these cells (CSF1^op/op^ mice) or by depletion using clodronate liposomes, then the terminal phase of elimination of *Mp* is impaired.

### Phagocytosis of *Mp in vitro* by macrophages

**Figure 5:**
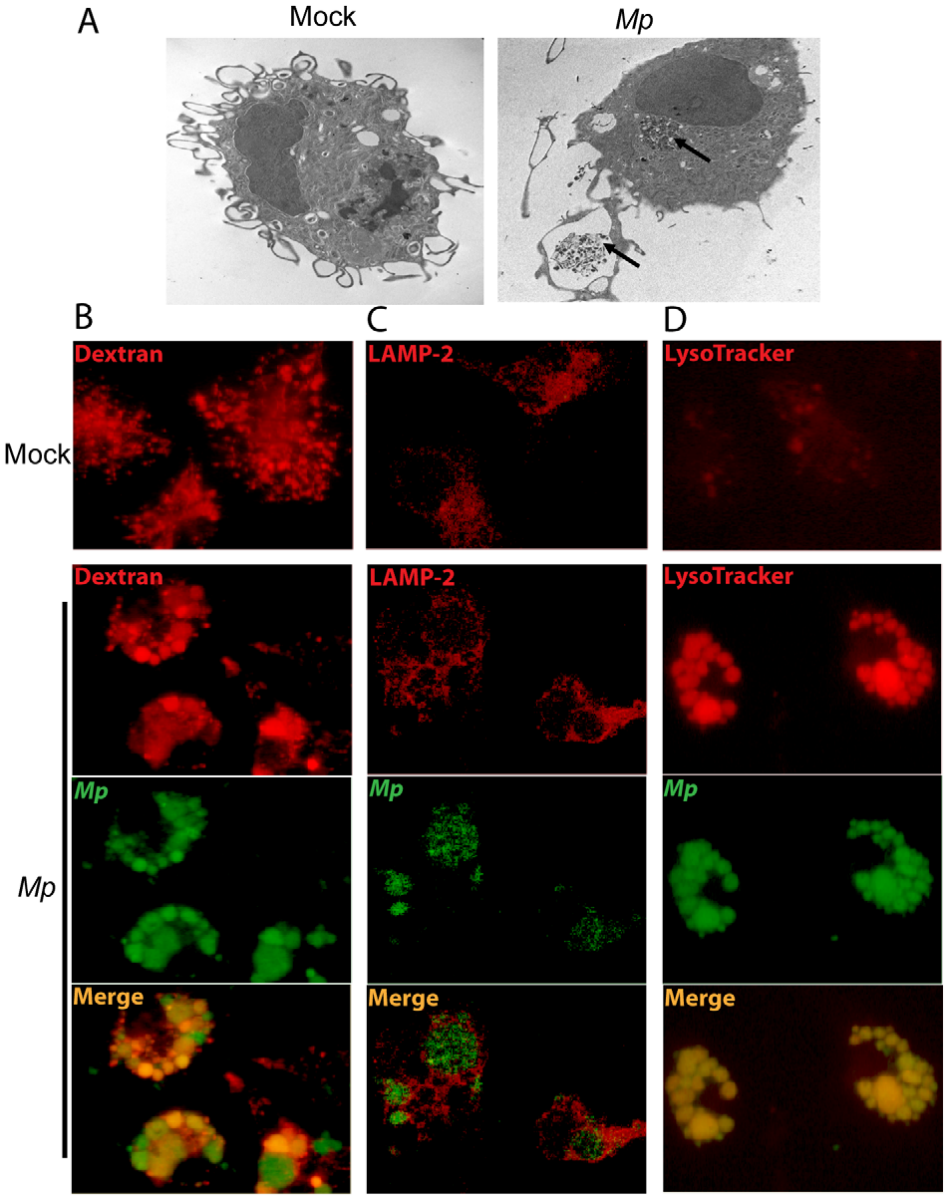
Macrophage phagocytosis of *Mp in vitro.* (A) BMM were infected with *Mp* for 1 h, and the cells were fixed with 2% paraformaldehyde and 2% glutaraldehyde prior to embedding for TEM. Internalized *Mp* are highlighted by the black arrows. BMM were pre-loaded with dextran to label endosomes (B), and stained with anti-LAMP-2 antibody to visualize lysosomes (C) or LysoTracker dye to visualize acidified compartments (D). (B-D) cultures were infected with *Mp* (MOI 100∶1) or mock infected with medium alone as indicated. All portions of these experiments were repeated at least two times, always with similar results.

Having established the importance of macrophages in the elimination of *Mp* from the lungs and airways, we next investigated how macrophages respond when cultured with the microbe *in vitro*. In order to visualize the encounter of macrophages with the microbe, we co-cultured *Mp* with adherent bone marrow-derived macrophages (BMM) from WT mice at a ratio of 100∶1 for 1 h, washed the adherent cells with PBS, and analyzed the cells by transmission electron microscopy. Cells cultured with *Mp* showed large clusters of the microbe in phagocytic vacuoles (Figure 5A, upper arrow) as well as clusters of bacteria apparently in the early phases of cellular uptake (Figure 5A, lower arrow). To analyze the role of phagocytosis in greater detail, we stained the cells with reagents specific for endosomes or lysosomes and with the LysoTracker dye, then analyzed the cells by fluorescence microscopy. To test for phagosome-endosome fusion, we preloaded the endosomes of BMM with fluorophore-conjugated dextran [32] and then incubated with *Mp* carrying a gene encoding enhanced yellow fluorescent protein (EYFP-*Mp*; see Materials and Methods) for 1 hr (Figure 5B). This experiment demonstrated co-localization of dextran with the majority of *Mp-*containing cell organelles, indicating that phagosome-endosome fusion had occurred.

To further examine the process of phagosome maturation, we stained the cells with the lysosome-specific marker LAMP-2 (Figure 5C). The addition of *Mp* to the cultured cells resulted in enhanced localization of LAMP-2 in close approximation to vacuoles that contained abundant *Mp,* showing the localization of *Mp* with a phago-lysosomal compartment. To investigate whether the *Mp*-containing vesicles were acidified, we cultured BMM with EYFP-*Mp* and then stained the cells with LysoTracker Red, an indicator of phagolysosome acidification (Figure 5D). Addition of *Mp* to the cultured BMM resulted in dramatically enhanced LysoTracker-dependent staining of phagolysosomal vacuoles, with co-localization of the intracellular *Mp* and the acid indicator dye. Thus, as early as 1 h after infection, *Mp* had been taken up by macrophages into a phagosomal compartment that fused with lysosomal elements to form an acidified phagolysosome.

### Cellular adhesive interaction is required for persistence of *Mp* in the murine respiratory tract but not for macrophage-dependent *Mp* clearance

To understand further the molecular mechanisms by which macrophages recognize invading *Mp*, we tested the importance of adhesive interactions for clearance of the microbe. The mycoplasmal P1 adhesin is an essential component of the mycoplasma attachment organelle by which the microbe adheres to the surface of host epithelial cells in the respiratory tract [12]. P1-deficient *Mp* loses its ability to attach to the host airway epithelium and expresses greatly reduced virulence [33]. This is manifested by accelerated clearance of P1-deficient *Mp* from the respiratory tract of WT mice (Figure S5). To test whether macrophages are required for clearance of P1-deficient *Mp*, we inoculated macrophage-depleted mice with P1-deficient *Mp* and determined the numbers of *Mp* present in the lungs at various subsequent time points. Depletion of macrophages resulted in prolongation of the persistence of the P1-deficient *Mp*, but they were still cleared more rapidly than WT *Mp* (Figure S5). Thus, P1-mediated interactions between *Mp* and the responding macrophage are not required for macrophage-dependent clearance of the microbe from the lungs and airways. These data suggest that, while P1-dependent *Mp*-host cell interactions contribute importantly to the ability of the microbe to persist in the respiratory tract, non-P1-dependent mechanisms are central to the interactions of *Mp* with host macrophages. We hypothesized, consequently, that macrophage PRR might contribute importantly to this response.

### Signaling through the adaptor protein MyD88 is essential for the macrophage response to *Mp* in the lungs

The TLR family is one of the best-characterized families of PRR. It has been shown to play a key role in the triggering of both the innate and the adaptive arms of the immune response [15], and serves as a bridge between innate and adaptive immunity [34], [35], [36]. MyD88 is the major adaptor molecule for signaling downstream of TLR [37]. Therefore, we tested the role of TLR-MyD88 signaling in the clearance of *Mp* from the lungs of mice.

**Figure 6:**
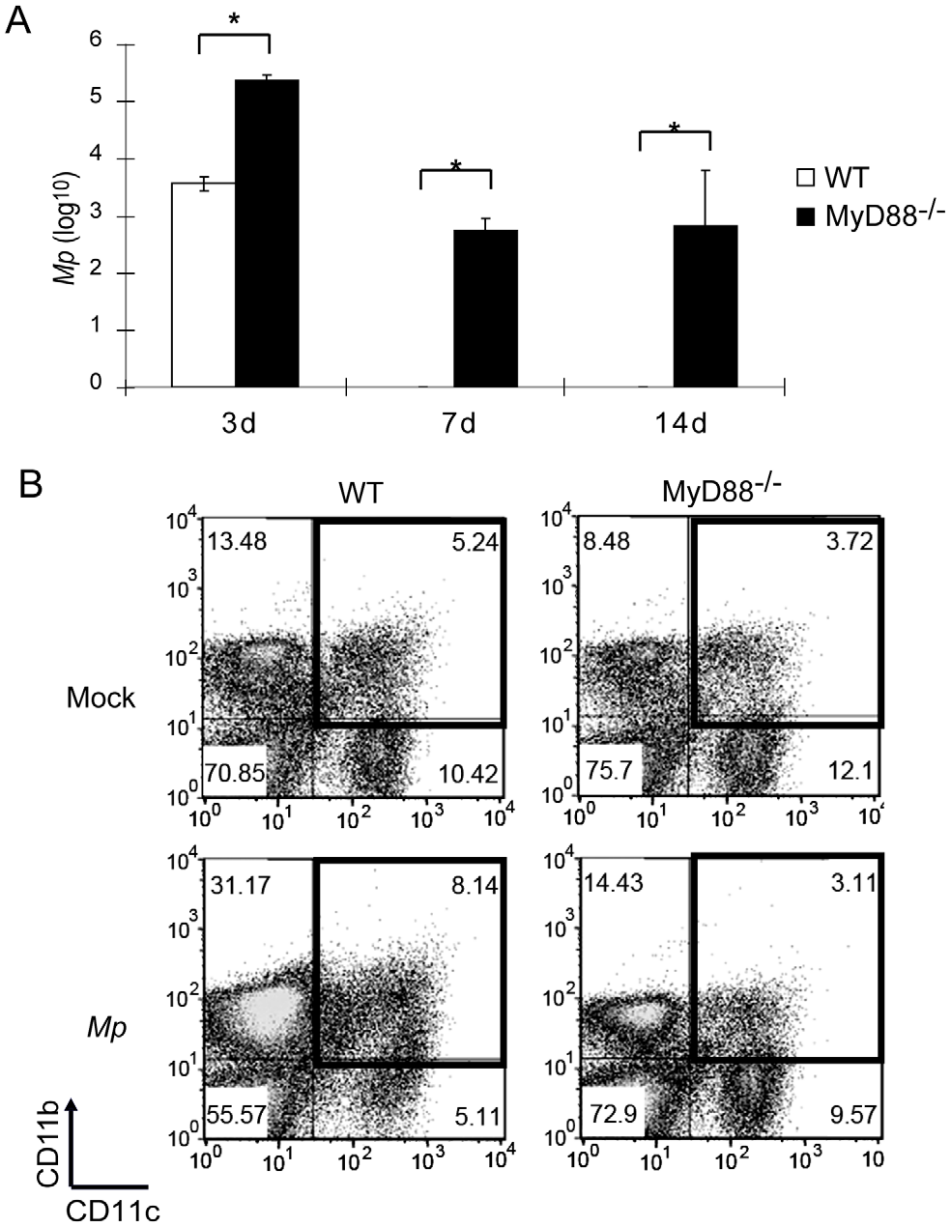
The macrophage response to *Mp* in mouse lung is MyD88-dependent. WT and MyD88^−/−^ mice were inoculated with *Mp*, and (A) the survival of *Mp* in the lungs was determined at the indicated times. (*p<0.05) (B) Total leukocytes were recovered from collagenase-digested lungs, and cells were stained with anti-CD11c and anti-CD11b prior to analysis by flow cytometry. Three independent experiments showed similar results. Data shown are means ± SEM (n = 4 or 5). *p<0.05.

Three days after inoculation with 4×10^6^ *Mp*, there were 10^5^ *Mp* remaining in the lungs of MyD88^−/−^ mice, compared to 10^3^ *Mp* in lungs of WT mice (Figure 6A). Seven days and 14 days after inoculation with *Mp*, the microbe was undetectable in the lungs of WT mice; in contrast, 10^2^-10^3^ *Mp* were detected in the lungs of MyD88^−/−^ mice (Figure 6A). This significant impairment in the elimination of *Mp* from the lungs of MyD88^−/−^ mice indicates that the signaling adaptor MyD88 is essential for the elimination of *Mp* from the murine respiratory tract and suggests that TLR signaling plays an important role in this process.

Because the TLR-MyD88 signaling pathway serves a major role in activation of macrophages, and because macrophages are critical for clearance of *Mp*, we tested whether MyD88 is required for recruitment into the lungs and activation of macrophages and monocyte/macrophages following inoculation with *Mp*. In naive WT mice, 5.2% of the total pulmonary cells were CD11c^+^CD11b^+^ macrophages (Figure 6B); one day after inoculation, the percentage of CD11c^+^CD11b^+^ cells was increased to 8.1% (Figure 6B), consisting of CD11C^hi^CD11b^lo^ and CD11c^lo^CD11b^hi^ subpopulations (Figure S4). The CD11c^hi^CD11b^lo^ subpopulation appeared to represent activated cells generated from the CD11c^+^CD11b^−^ subpopulation of resident lung macrophages (Figure 6B). The other population was CD11c^lo^CD11b^hi^, and appeared to represent newly recruited monocyte/macrophages because they were absent from mice that had been treated with clodronate liposomes administered i.v., but not i.n. (Figure S4). In contrast to leukocytes detected in the lungs of WT mice, CD11c^+^CD11b^+^ macrophages were not increased in the lungs of *Mp*-treated MyD88^−/−^ animals (Figure 6B). Rather, it appeared both that the CD11c^+^CD11b^−^ lung resident macrophages of MyD88^−/−^ mice were not activated following treatment with *Mp*, and that the recruitment of monocyte/macrophages from the periphery into the lungs was impaired in MyD88^−/−^ mice (Figure 6B).

### *A*ctivation of macrophage NFκB by *Mp* depends on MyD88

**Figure 7:**
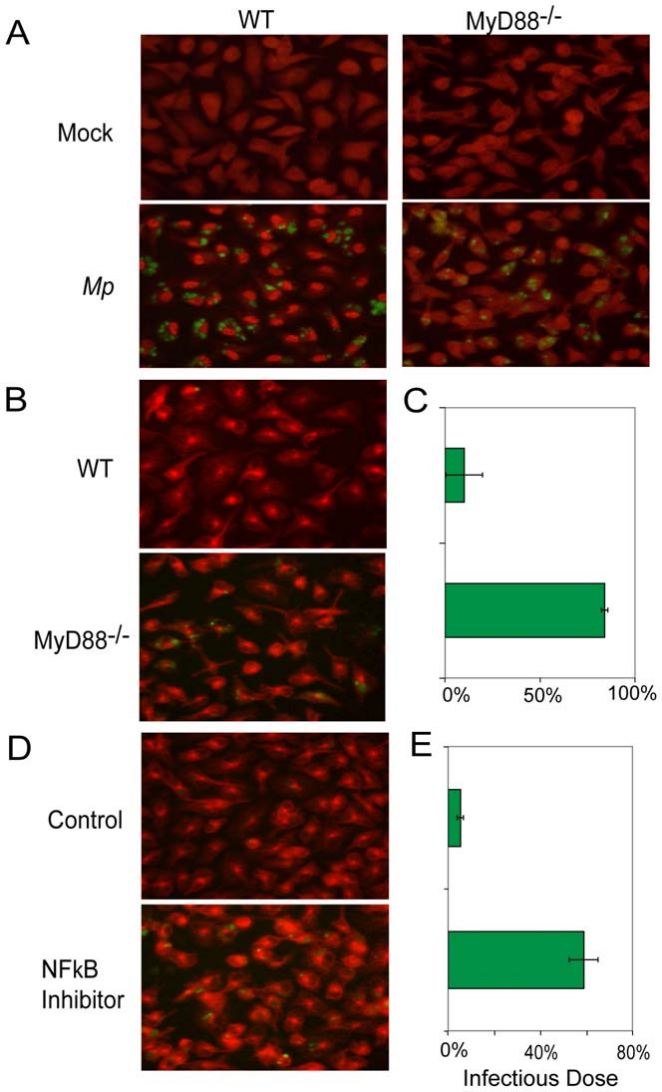
MyD88-NFκB signaling is essential for macrophages to eliminate *Mp*. (A), (B) and (C), WT and MyD88^−/−^ BMM were infected with EYFP-*Mp* or mock infected. (A) One hour after infection with EYFP-*Mp* (green), BMM were stained with anti-NFκB (p65) (red) to determine the subcellular location of this transcription factor. (B) BMM were stained with anti-α-tubulin (red) to highlight cell morphology. The survival of EYFP-*Mp* (green) was estimated by microscopy, and (C) the survival of EYFP-*Mp* was analyzed quantitatively by real-time PCR. (D) and (E), WT BMM were pretreated with the inhibitor of NFκB activation or with the diluent control (DMSO) for 1 h prior *Mp* infection. Eight hours after infection, BMM were stained with anti-α–tubulin (red) to show cell morphology and surviving EYFP-*Mp* (green) (D). The survival of EYFP-*Mp* in the cultures shown in (D) was determined by real-time PCR (E). Data shown are representative of two replicate experiments and are means ± SEM (n = 3).

In order to examine whether MyD88 has a direct role in the macrophage response to *M*p, we analyzed the activation of NFκB, one of the major downstream transcription factors activated by TLR-MyD88 signaling, in WT or MyD88^−/−^ BMM treated *in vitro* with EYFP-*Mp*. Before infection with *Mp* or following mock infection, the majority of NFκB was distributed diffusely in the cytoplasm in both WT and MyD88^−/−^ BMM (Figure 7A). One hour after infection of WT BMM with *Mp*, NFκB had largely translocated into the nuclear compartment (Figure 7A), whereas it remained in the cytoplasm in BMM from MyD88^−/−^ mice (Figure 7A). In order to assess the activation of NFκB quantitatively in *Mp* infected BMM from WT and MyD88^−/−^ mice, we used an ELISA that only detected the activated form of this transcription factor by virtue of its ability to recognize its specific DNA target sequence. Before infection with *Mp* or mock infection, BMM from WT or MyD88^−/−^ mice have similar low levels of activated NFκB in the total cell extracts (Figure S6A). One hour after *Mp* infection, there was a significantly increased level of NFκB activation in WT BMM, but not in MyD88^−/−^ BMM (Figure S6A).

We then tested in BMM and in F4/80^+^ macrophages purified from lungs of WT and MyD88^−/−^ mice the impact of MyD88-deficiency on the mRNA expression of pro-inflammatory cytokines and chemokines that depend on NFκB for their induction. The mRNA expression of IL-1β, IL-6, TNF, and MIP-2 were up-regulated in *Mp*-infected WT BMM and purified macrophages at all time points tested; in contrast, there was only very low level expression of these pro-inflammatory genes in bone marrow-derived and lung macrophages of MyD88^−/−^ mice following *Mp* infection (Figure S6B and data not shown). These data indicated that the MyD88-NFκB pathway responds rapidly to *Mp*, and suggested that this pathway may contribute importantly to the elimination of *Mp* by macrophages. They showed, further, that bone marrow-derived macrophages respond similarly to primary lung macrophages following contact with *Mp.*

### Functional defect in elimination of *Mp* by MyD88-NFκB deficient macrophages

To test the hypothesis that MyD88-NFκB signaling contributes to the ability of macrophages to clear *Mp*, we analyzed the survival of EYFP-*Mp* in cultures of WT and MyD88^−/−^ BMM using both semi-quantitative fluorescence microscopy and real-time PCR. One hour after addition of EYFP-*Mp* to BMM cultures, clusters of internalized bacteria were visible in both the WT and the MyD88^−/−^ cells (Figure 7A). By 8 h after infection of WT BMM cultures with EYFP-*Mp*, there were almost no visible bacteria remaining (Figure 7B). In contrast, significant numbers of EYFP-*Mp* were easily detected at 8 h in cultures of MyD88^−/−^ BMM (Figure 7B). Analysis of the numbers of cell-associated *Mp* by real-time PCR at 8 h after infection showed that approximately 10% of the original inoculum remained in the WT BMM culture (Figure 7C), compared to approximately 80% of the original inoculum in the MyD88^−/−^ BMM culture (Figure 7C).

To test whether activation of NFκB, a major transcription factor downstream of MyD88, is required for the elimination of *Mp* by BMM, we treated BMM cultures with an inhibitor of NFκB activation for 1 hour prior to infection with EYFP-*Mp*. Eight hours after infection, there were only a few small clusters of cell-associated EYFP-*Mp* visible in the BMM cultured with the vehicle control, whereas a substantial amount of clustered EYFP-*Mp* were seen in the BMM cultures that had been treated with the inhibitor of NFκB activation (Figure 7D). Analysis by real-time PCR demonstrated that less than 10% of the original inoculum of *Mp* remained in the control BMM cultures, compared to approximately 60% of the original inoculum in the cultures of BMM that had been treated with the NFκB inhibitor (Figure 7E). These data underscore the critical role that the MyD88-NFκB pathway plays in the macrophage-mediated elimination of *Mp*.

## Discussion

*Mycoplasma pneumoniae* (*Mp*) has been isolated and cultivated since the early 1960s and remains a common cause of community-acquired pneumonia. Over the past 17 years, several groups have observed that asthmatic subjects are at increased risk for colonization or productive infection with *Mp* compared to non-asthmatic controls [5], [38], [39], [40]; however, there has been only limited progress in understanding the pathogenic mechanisms that underlie *Mp* infections and the immunological mechanisms of their association with asthma. Our study has focused on identifying the cellular and molecular mechanisms that govern protective host responses to *Mp* in an experimental rodent model in order to permit development of therapies that interrupt the synergy between *Mp* and atopy in the expression of the asthmatic phenotype.

The finding of *Mp* in the airways of substantial numbers of individuals with asthma suggests either that the presence of *Mp* in the airways contributes to exacerbations of asthma symptoms in these asthmatic patients or that asthmatic inflammation alters the clearance of *Mp* from the airways, or both. Supporting a role for *Mp* in asthma exacerbations, one study showed that treatment with clarithromycin, an antibiotic that suppresses the growth of *Mp* resulted in significantly reduced asthma symptoms and improved lung function in *Mp*-positive asthmatic subjects, but not in *Mp*-negative subjects [8]. A recent follow-up study was not able to replicate this finding because the number of asthmatics PCR positive for *Mp* was too low (only 13%) [41]. The authors noted, however, that the study design may have impaired the detection of *Mp* since it included a run-in period with inhaled corticosteroids, a class of drugs that has been shown in a murine infection model to impair the growth of *Mp* in lung tissue [42]. In this group's previous studies [7] the chances that an asthmatic with a positive PCR test for *Mp* was not taking corticosteroids nearly reached statistical significance. This result underscores the importance of addressing the ways in which *Mp* may contribute to or modulate asthmatic inflammation and the mechanisms by which the host normally eliminates the microbe from the respiratory tract.

*Mycoplasma pulmonis* (*M. pulmonis*), a natural pathogen for rodents, has been widely utilized in mice and rats as a model system to mimic *M. pneumoniae*-induced pneumonia in humans. These studies have established several key features of the host response to *M. pulmonis*; however, much remains to be learned, and differences in the way the host handles the different mycoplasma species can be anticipated. Both alveolar macrophages and mast cells have been linked with the clearance of *M. pulmonis* in mice. Depletion of alveolar macrophages has been shown to cause exacerbation of respiratory mycoplasmosis in the *M. pulmonis*-resistant C57BL/6 mouse strain, although interestingly not in the *M. pulmonis*-susceptible strain C3H [43], [44]. These findings emphasize the role of macrophage lineage cells in effective host responsiveness to mycoplasma. These investigators have gone on to demonstrate that two of the mechanisms that alveolar macrophage utilize to eliminate *M. pulmonis* depend on normal levels of Surfactant protein A [45] and nitric oxide [46].

Additionally, mice deficient in mast cells due to their carriage of the Kit^wsh/wsh^ mutation showed more severe weight loss and higher-grade pneumonia compared to WT mice infected with *M. pulmonis*. This phenotype was associated with a greater burden of mycoplasma in the lungs at early time points after experimental infection [47]. Although these investigators did not assess the effect of restoring mast cells in the Kit mutant mice, their findings suggested a protective role of mast cells in the host response to *M. pulmonis*.

Although *Mp* is not a natural pathogen in rodents, prior studies have shown that i.n. inoculation with *Mp* induces airway inflammation in mice [48]. Consistent with this, our data demonstrate that the microbe elicits a reproducible cellular inflammatory response in the lungs and airways of this experimental host. The inflammatory response is observed within several hours after inoculation with the microbe, and is dominated by the entry into the lungs of a variety of myeloid lineage cells, including neutrophils, macrophages, and dendritic cells (Figure 2). In spite of the rapid mobilization of these inflammatory cells, the microbe is able to survive for an extended period of time after inoculation, with complete clearance from the airway requiring 1 to 4 weeks, depending on the mouse strain being studied (Figure 1). Although full clearance requires a week or more and is associated with the development of circulating anti-*Mp* antibodies [49], the participation of Rag1-dependent adaptive immunity is not required (Figure S2).

These observations indicate that the primary mechanisms protecting the naïve murine host from *Mp* are components of the innate immune system. Prior studies in both humans and mice have focused on the roles of pro-inflammatory cytokines and chemokines, and have demonstrated increased production of IL-1β, IL-6, IL-8, IL-12, and KC in the respiratory tract [10], [48].

Consistent with the elevated expression of IL-1β, IL-6, IL-8/KC, and IL-12 after airway instillation of *Mp*, the microbe triggers rapid recruitment of a large number of neutrophils and macrophages into the lungs and airways (Figures 2C and 2D). Both of these cell types generally assist in the clearance of bacterial pathogens from the lungs of mice and humans [50], [51]. In the case of mycoplasma, however, prior data from Simberkoff and Elsbach has shown that *in vitro* co-incubation of mycoplasma with neutrophils had little impact on the viability of the microbe, suggesting that neutrophils are unlikely to participate in the killing and elimination of this microbe *in vivo* [52]. Thus, even though neutrophils are rapidly recruited in large numbers after airway inoculation with *Mp*, depletion of these cells by treatment with the anti-Gr-1 antibodies RB6-8C5 (Figure 3) or NIMP-R14 (data not shown) did not slow the clearance of the microbe from the lungs and airways. In fact, in some experiments, depletion of Gr-1^+^ cells resulted in a trend towards more rapid clearance of *Mp* from the airways (for example, Figure 3B), suggesting that neutrophils or other Gr-1^+^ cells may play an anti-inflammatory role in the host response to this microbe, modestly slowing its elimination. Regardless of the possible anti-inflammatory functions of neutrophils in the anti-mycoplasma response, other components of the innate immune system must be central in the elimination of the microbe.

Because NK cells, NK-T cells and mast cells produce mediators that can potently activate antibacterial host defenses [53], [54], [55], we tested whether these cells contribute importantly in the clearance of *Mp* by inoculating mice deficient in one or more of these lineages with the microbe. Mice that had been injected with anti-NK1.1 antibody (to deplete NK cells and NK-T cells), IL-15^−/−^ mice (which are congenitally deficient in NK cells), and CD1d1^−/−^ mice (deficient in NK-T cells) showed clearance of *Mp* from the airways that was indistinguishable from WT mice (data not shown), indicating no obligatory role for these cell lineages in elimination of the pathogen. Additionally, congenitally mast cell-deficient c-kit^wsh/wsh^ mice showed no impairment of *Mp* clearance (Figure S7). These latter findings are of interest in the context of the findings of Xu et al. [47] who demonstrated that clearance of *M. pulmonis* is impaired in the c-kit^wsh/wsh^ strain. The differences in the impact of this mutation on the ability of mice to clear *Mp* and *M. pulmonis* suggest that there may be microbial strain-specific aspects of the host response to this family of organisms.

Like neutrophils, macrophage lineage cells are also prominently recruited to the lungs within several hours after inoculation of *Mp* into the airways (Figures 2C, 2D and 2E). Experiments using pharmacologic depletion of macrophages (by treatment with clodronate liposomes; Figure 4B) or mice with congenital deficiency of macrophages (the CSF1^op/op^ mouse strain; Figure 4C) showed that macrophages were indeed critical for the clearance of *Mp*. Interestingly, both of these approaches showed that the clearance of *Mp* occurred in at least two phases – an early macrophage-independent phase extending until approximately 3 d after i.n. inoculation with the microbe, and a late, macrophage-dependent phase, from day 3 to final eradication of the microbe. The initial macrophage-independent phase involves clearance of greater than 99% of the *Mp* inoculum. The nature of the innate mechanisms that govern this portion of the response remain undefined. As discussed above, neither the early nor the late phases of *Mp* clearance requires NK cell or neutrophil function. Using analysis of gene-targeted mice, we have also shown that neither the third component of complement (utilizing C3^−/−^ mice) nor the free radical mediator nitric oxide produced by iNOS (using iNOS^−/−^ mice) is essential for clearance of *Mp* (Figure S8). The reasons that iNOS is not required for clearance of *Mp* from the lungs of mice, but is required for the normal airway response to *M. pulmonis* [45] remain to be investigated.

In considering the potential molecular mechanisms by which macrophages govern the elimination of *Mp* from the lungs and airways, we noted that the rate of clearance of this microbe from the respiratory tract is substantially faster from C57BL/6 mice compared to BALB/c mice. This appeared to include differences in both the early phase of the response and the late phase. C57BL/6 and BALB/c mice are known to differ in their production of several key cytokines. Macrophages from C57BL/6 mice respond to stimulation by LPS with expression of higher quantities of IFN-γ and IL-12 compared to macrophages from BALB/c mice [56]. This and other studies suggest that C57BL/6 mice favor Th1 responses, whereas BALB/c mice favor Th2-type responses. Our data are consistent with host defense mechanisms associated with Th1-type responses participating importantly in the clearance of *Mp* from the airways. Interestingly, Salvatore et al. reported that mice deficient in IL-12 P35 showed accelerated clearance of *Mp* from the airways [18], suggesting that the prototypic Th1 response itself is not responsible for *Mp* clearance. Consistent with this is our finding using Rag1^−/−^ mice that functional lymphocytes are not required for clearance (Figure S2).

Studies both *in vivo*, analyzing clearance from the lungs (Figure 6A), and *in vitro*, analyzing the handling of *Mp* by macrophages (Figure 7A), showed a pivotal role for the MyD88 adapter protein in clearance of *Mp*. MyD88 appears to affect both the early, macrophage-independent phase of microbial clearance and the late, macrophage-dependent clearance phase. This latter observation is consistent with the requirement for My88 for normal clearance of the microbe by *Mp* cultured *in vitro*. The MyD88-interacting receptor that governs this effect on clearance of *Mp* has not been defined. Using IL-1β^−/−^ and type I IL-1 receptor^−/−^ mice, we have excluded an essential role of the IL-1 axis in the clearance of the microbe (data not shown). Studies are underway to test the role of IL-18 and individual TLR in the clearance of *Mp*. Importantly, it has been identified that *Mp*-derived lipoproteins induce expression of the inflammatory cytokine TNF in macrophages through TLR2 paired with TLR1 or TLR6 [57]. Thus, TLR2 might be a candidate receptor that plays a role in the macrophage response to *Mp*.

In all settings that we have analyzed, BMM functioned similarly to macrophages purified from the lungs of mice (Figures S6B and S6C, and data not shown). Incubation of BMM with fluorescently labeled *Mp* followed by analysis both by transmission electron microscopy and by confocal fluorescence microscopy indicated that elimination of *Mp* is associated with their phagocytosis into LAMP-2-associated, acidified intracellular vacuoles. While we cannot rule out some degree of extracellular killing of the microbe, our data are consistent with the majority of the killing of *Mp* occurring in the phagolysosomal system. The central role of NFκB in disposal of the microbe suggests that pharmacological manipulations that altered the function of this transcription factor could have a profound effect on clearance of this organism.

Given that MyD88-dependent and NFκB-dependent pathways are involved in the activation of neutrophils [58], it is of considerable interest that neutrophils appear not to be engaged in the clearance of *Mp* from the lungs and airways. Additional studies will be required to determine the requirements for phagocytosis of this atypical microbe that lacks a cell wall. The fact that neutrophils are not required for the clearance of *Mp* from naïve mice does not exclude a potentially significant role for neutrophils in the clearance of opsonized *Mp* as might be encountered in mice that had established a robust anti-*Mp* antibody response.

The prominent roles of both macrophages and MyD88 in the anti-*Mp* response establish the key role of innate immune function in the elimination of this microbe. One might anticipate that deficiencies of MyD88 or TLR function could impair clearance of *Mp* and lead to persistence of asthma symptoms associated with chronic carriage of the organism as observed by Kraft et al. [40]. Such a potential relationship between TLR function and airway *Mp* may be particularly relevant in light of the findings by Kormann et al. that polymorphic variants of TLRs 1, 2, and 6 contribute to susceptibility and resistance to asthma in children [59].

Macrophages and TLRs should be studied further in asthmatic individuals who are colonized or infected by *Mp*. Additional studies should address the potential impact of asthmatic inflammation on the ability of pulmonary macrophages to respond to and eliminate this microbe. Furthermore, maneuvers that potentiate macrophage anti-mycoplasma function without up-regulating the proinflammatory activities of these cells might afford ways to improve, in an antibiotic-sparing fashion, asthma symptoms in individuals who are carrying this microbe.

## Materials and Methods

### Ethics statement

This study was carried out in strict accordance with the recommendations of the Guide for the Care and Use of Laboratory Animals of the National Institutes of Health. The protocols for all experiments using vertebrate animals were approved by the Institutional Animal Care and Use Committee of the University of Alabama at Birmingham (Protocol number 091209016 under Institutional Animal Assurance Number A-3255-01).

### Mice

C57BL/6, BALB/c, Rag1^−/−^, Csf1^op/op^, iNOS^−/−^, and c-kit^wsh/wsh^ mice were obtained from The Jackson Laboratory (Bar Harbor, ME). C3^−/−^ mice were from Dr. Harvey Colten [60] and were kindly provided by Dr. Scott Barnum (University of Alabama at Birmingham (UAB)). MyD88^−/−^ mice [61] were from Dr. Shizuo Akira (Osaka University, Osaka, Japan) and were kindly provided by Dr. Suzanne Michalek (UAB). All mice were used between 6 to12 weeks of age and were kept in micro-isolator cages in the specific pathogen-free Animal Resources Program facility at UAB.

### Reagents and antibodies

Anti-Ly6C-FITC (Abcam, Cambridge, MA), anti-Ly6G-PE, anti-CD11b-APC-Cy7, anti-CD11c-PE-Cy7 (BD Bioscience, San Jose, CA), anti-F4/80-PE-Alexa 647 (AbD Serotec, Raleigh, NC), anti-Gr-1- APC (Invitrogen, Carlsbad, CA), rabbit anti-NFκB p65 (Santa Cruz Biotechnology, Santa Cruz, CA), mouse anti-α-tubulin (Invitrogen), and goat anti-rabbit IgG-Rhodamine Red X (RRX) and goat anti-mouse IgG-RRX (Jackson ImmunoResearch Laboratory, West Grove, PA) were used for flow cytometry and immunofluorescence microscopy. The inhibitor of NFκB activation [62], [63] (Calbiochem, San Diego, CA) and DMSO (Sigma-Aldrich, St. Louis, MO) alone as control were used *in vitro* to assess the role of activated NFκB in the host response to *Mp*.

### Culture of BMM

For preparation of bone marrow-derived macrophages (BMM), total bone marrow cells were recovered aseptically from the femurs of euthanized adult mice and filtered through a 40-µm cell strainer to remove undispersed cells and tissue debris. Approximately 2×10^6^ bone marrow cells were cultured in a 10 cm Petri dish with 10 ml of RPMI 1640 medium plus 10% FBS, 2 mM L-glutamine, 1X Pen/Strep (complete RPMI medium) and 20% L929 cell conditioned medium. On d 5, an additional 5 ml of complete RPMI medium were added, and the following day the adherent cells were harvested by trypsinization and re-plated in complete RPMI medium at a density of 1.5×10^6^ cells per well of a 6-well plate or 7.5×10^5^ cells per well of a 12 well plate or per well of a 2 well chamber slide. BMM were rested for 1 day before infection with *Mp*.

### Infection with *Mp*

For analysis of the clearance of *Mp in vivo*, mice were anesthetized lightly with isoflurane, and then inoculated intranasally (i.n.) with 4×10^6^ WT *Mp* strain M129 (ATCC 29342) in 40 µl of SP4 medium. Mock inoculations were with SP4 medium alone. At various times after inoculation, whole lungs were harvested for extraction of total RNA or preparation of cell suspensions. For *in vitro* studies, BMM were infected with WT *Mp* or with a strain of *Mp* that was deficient in the P30 adhesin and restored to WT by transformation with a recombinant transposon carrying a fusion gene encoding P30 and EYFP (EYFP-*Mp*) [64]. Infections of cultures of BMM *in vitro* were at a 100∶1 multiplicity of infection (MOI). Controls were mock infected with complete RPMI 1640 medium alone. For transmission electron microscopy and for analysis of translocation of NFκB, BMM were analyzed 1 hour after infection with *Mp in vitro*. For determining the *Mp*-induced expression of RNA encoding cytokines and chemokines, BMM were harvested 3 hours after infection. For investigating the elimination of *Mp* from BMM cultures, BMM were harvested 8 hours after infection.

### Detection of *Mp* by bacterial culture

Numbers of viable *M. pneumoniae* in the lungs and airways of experimentally infected mice were determined by a modification of the procedure of Cartner et al. [43]. Whole lungs were harvested by dissection from the main stem bronchi and placed in a small volume of SP4 medium at room temperature. The lung tissue was finely minced in 2 ml SP4 broth and vortexed for 30 sec. Tenfold dilutions were prepared and 20 µl aliquots were plated on SP4 agar and incubated at 37°C in 5% CO_2_ for 7 to 14 days until colonies were visible. Colonies were counted under a dissecting microscope. In addition, the 10-fold dilutions were incubated at 37°C for 14 days, then observed for development of a color change indicating growth. All samples were processed and analyzed in a double blind fashion.

### Detection of *Mp* by real-time PCR

Whole lungs were harvested by dissection from the main stem bronchi, stored in the RNA stabilizing solution RNAlater (Ambion, Austin, TX), and kept at −20°C prior to isolation of total cellular RNA. Cultured macrophages were directly dissolved in RLT lysis buffer (Qiagen, Valencia, CA). Then total RNA was extracted from whole lungs or from macrophage lysates using the RNeasy kit (Qiagen), and reverse transcribed into cDNA using the SuperScript III RTS First-Strand cDNA Synthesis Kit (Invitrogen). The numbers of *Mp* in lung samples were determined by Taqman real-time PCR using a set of primers and probe that were specific for the 16S rRNA of *Mp*. We used the 16S rRNA as the target for quantifying *Mp* because this RNA is present in >1,000 copies per bacterium, enhancing the sensitivity of the assay [65]. The primer sequences providing specificity for *Mp* were 5′-GGA CCT GCA AGG GTT CGT T-3′ (forward primer), 5′-AGT TGG TGG GGT AAC GGC C-3′ (reverse primer), and 5′-/56-FAM/-TTG ATG AGG GTG CGC CAT ATC AGC T-/3BHQ-1/-3′ (probe). We used the murine glyceraldehyde-3-phosphate dehydrogenase (GAPDH) gene as a control for normalizing the quantity of total lung RNA recovered in each experiment. The GAPDH primers were 5′-TCC ATG ACA ACT TTG GCA TTG-3′ (forward primer), 5′-CAG TCT TCT GGG TGG CAG TGA-3′ (reverse primer), and 5′-/5TexEd-XN/-AGG GCT CAT GAC CAC AGT CCA TGC C-/3BHQ-2/-3′ (probe) (all purchased from Integrated DNA Technologies (IDT), Coralville, IA).

### Flow cytometry

Freshly harvested lungs were minced and digested with Collagenase B (2 mg/ml) and DNase I (0.02 mg/ml) for 30 minutes at 37°C. Single cell suspensions were filtered through a 40-µm cell strainer and then fixed with 2% paraformaldehyde prior to staining with fluorescently conjugated Abs, washing and analysis using an LSRII flow cytometer (BD Biosciences) using FlowJo software (Tree Star, Inc., Portland, OR).

### Depletion of leukocyte populations *in vivo*

For depletion of neutrophils, mice were injected i.v. with 100 µg of the rat anti-murine Gr-1 mAb (RB6-8C5; kindly provided by Emil R. Unanue, Washington University, St. Louis, MO) or rat IgG (Sigma-Aldrich) in 100 µl of PBS. Two days after injection with the depleting antibody, mice were inoculated i.n. with *Mp*. Depletion of macrophages was performed using liposomes containing dichloromethylene bisphosphonate (clodronate; [66]). Clodronate was a gift of Roche (Mannheim, Germany). It was encapsulated in liposomes as previously described [67]. Phosphatidylcholine (LIPOID E PC) was obtained from Lipoid GmbH. Cholesterol was purchased from Sigma-Aldrich. Mice were injected i.n. with 50 µl and i.p. with 100 µl of clodronate- or PBS-containing liposomes 24 h prior to inoculation with *Mp*.

### Analysis of NFκB localization and cell morphology by immunofluorescence microscopy

At various times after infection with EYFP-*Mp*, BMM cultured in chamber slides were washed twice with PBS, fixed with 2% paraformaldehyde for 20 min at RT, and permeabilized with CSK buffer (10 mM PIPES, pH 6.8, 50 mM NaCl, 300 mM sucrose, 3 mM MgCl_2_, 0.05% Triton X-100) for 5 min at RT. Translocation of NFκB from the cytoplasm to the nucleus was detected by staining with rabbit anti-murine NFκB Ab followed by RRX labeled goat anti-rabbit IgG. Activation of the BMM cytoskeleton was assessed by staining with mouse anti-murine α-tubulin mAb followed by RRX labeled goat anti-mouse IgG.

### Statistical analyses

Statistical analyses were performed by two-tailed equal variant Student's *t* test. A *p* value <0.05 was considered statistically significant.

## Supporting Information

Figure S1 Comparison of *Mp* quantification in lungs by real-time PCR and bacterial culture. WT mice were inoculated intranasally with 4×10^6^ *Mp* at d0. At d1, d3 and d5 after inoculation, mice were sacrificed, and the whole lungs were harvested for RNA extraction (4 mice) or bacterial culture (4 different mice) for quantification of *Mp*. Data shown are means ± SEM (n = 4).
(0.48 MB EPS)

Figure S2 Adaptive immune responses are not required for the elimination of *Mp* from the lungs of naive mice. WT (open bars) and Rag1^−/−^ (filled bars) mice were inoculated intranasally with 4×10^6^ *Mp*. At the indicated times, total lung RNA was harvested and the numbers of *Mp* were measured by real-time PCR. Data shown are means ± SEM (n = 4 or 5). This experiment was repeated with similar results.
(0.29 MB EPS)

Figure S3 Mice with dysfunctional neutrophils show no defect in the elimination of *Mp* from the lungs. WT (blue bars) and CD11b^−/−^ (purple bars) mice were inoculated intranasally with 4×10^6^ *Mp*. Five days after inoculation, total lung RNA was harvested and the numbers of *Mp* were measured by real-time PCR. This experiment was repeated with similar results. Data shown are means ± SEM (n = 5).
(0.47 MB EPS)

Figure S4 Two major populations of macrophages found in the lungs of mice following i.n. inoculation with *Mp* can be depleted differentially by treatment with clodronate liposomes. Mice were injected with clodronate liposomes or control (PBS) liposomes via the i.n. and i.p. routes one day prior to inoculation with *Mp*. On d1 after inoculation with *Mp*, lungs were harvested and digested into single cell suspensions, and then lung leukocytes were stained with anti-CD11c and anti-CD11b and analyzed by FACS. (A) Mice received i.n. and i.p. injection of control (PBS) liposomes. (B) Mice were pretreated with clodronate liposomes via the i.n. route only. (C) Mice were injected with clodronate liposomes by both the i.n. and i.p. routes. R1 (green box) represented CD11c^hi^CD11b^lo^ macrophages (activated resident pulmonary macrophages), and R2 (orange box) showed CD11c^lo^CD11b^hi^ macrophages (recruited peripheral macrophages). All parts of this experiment were repeated at least twice with similar results. Data shown are means ± SEM (n = 5).
(0.69 MB EPS)

Figure S5 The clearance of P1-deficient *Mp* is macrophage-dependent. Mice were treated i.n. and i.p. with clodronate liposomes to deplete macrophages, or with control (PBS) liposomes at day -1, followed by i.n. inoculation with P1-deficient *Mp* at day 0. At various times after inoculation with *Mp*, lungs were harvested and total RNA was extracted to determine the numbers of *Mp* by real-time PCR. (* p<0.05). This experiment was repeated one time with similar results. Data shown are means ± SEM (n = 5).
(0.33 MB EPS)

Figure S6 MyD88 signaling is essential for activation of NFκB and mRNA expression of pro-inflammatory genes in the macrophage response to *Mp*. BMM derived from wild type or MyD88^−/−^ mice were infected with *Mp* (MOI 100∶1), or mock infected as control. (A) One hour after infection, BMM were harvested and lysed with lysis buffer containing a phosphatase inhibitor. Five µg of total cell lysate were used for TransAM transcription factor ELISA (Actif Motif) to detect the activated form of NFκB. (B) BMM and (C) lung macrophages, total RNA was harvested at 0 h, 2 h, 4 h, 6 h and 8 h after *Mp* infection, and the mRNA expression of the pro-inflammatory genes TNF, IL-6, and MIP-2 was analyzed by real-time PCR. The mRNA expression levels of pro-inflammatory genes were compared to the levels in macrophages from WT mice without *Mp* infection (0 h). This experiment was repeated one time with similar results. Data shown are means ± SEM (n = 3 for BMM; n = 1 for primary lung macrophages).
(0.42 MB EPS)

Figure S7 The clearance of *Mp* from the airways of mice is independent of mast cells. WT (open bars) and c-kit^wsh/wsh^ (filled bars) mice were inoculated intranasally with 4×10^6^ *Mp*. At the indicated times, total lung RNA was harvested and the numbers of *Mp* were measured by real-time PCR. This experiment was repeated one time with similar results. Data shown are means ± SEM (n = 5).
(0.43 MB EPS)

Figure S8 The clearnce of *Mp* from the airways of mice is independent of C3 or nitric oxide produced by iNOS. (A) WT (open bars) and C3^−/−^ (filled bars) mice and (B) WT (open bars) and iNOS^−/−^ (filled bars) mice were inoculated intranasally with 4×10^6^ *Mp*. At the indicated times, total lung RNA was harvested and the numbers of *Mp* were measured by real-time PCR. Data shown are means ± SEM (n = 5).
(0.49 MB EPS)
